# Methodologies of Care: A Multimodal, Participatory Research Approach with Vulnerable Families Among South African Communities

**DOI:** 10.3390/mps9010011

**Published:** 2026-01-13

**Authors:** James Reid, Chanté Johannes, Shenaaz Wareley, Collen Ngadhi, Avukonke Nginase, Katerina Demetriou, Nicolette V. Roman

**Affiliations:** 1 School of Education, University of Huddersfield, Huddersfield HD1 3DH, UK; j.reid@hud.ac.uk; 2 Centre for Interdisciplinary Studies of Children, Families, and Society, University of the Western Cape, Cape Town 7535, South Africa; 3814572@myuwc.ac.za (S.W.); 4399086@myuwc.ac.za (C.N.); 4024125@myuwc.ac.za (A.N.); 4405978@myuwc.ac.za (K.D.); nroman@uwc.ac.za (N.V.R.)

**Keywords:** care, creative, multimodal, participatory research, vulnerable families

## Abstract

Multimodal methods provide valuable opportunities within Participatory Action Research (PAR), to foster meaningful participation, and amplify marginalized voices. However, conventional research approaches have not always adequately captured the complex realities of the lived experiences of families, and multimodal techniques have remained underutilized for the exploration of such experiences. This study aimed to explore the use of creative multimodal methods, within a PAR framework, grounded in care among vulnerable South African families. A qualitative design was adopted, incorporating Human-centered Design principles, within a PAR approach. The participants were recruited from the Saldanha Bay Municipality area (*n* = 70), as well as Mitchells Plain (*n* = 59). The multimodal methodology included Draw-and-Tell, painting, object and photo elicitation, I-Poems, and LEGO^®^-based activities. Data were annotated and transcribed verbatim, followed by thematic analysis. A total of 42 participants contributed towards the validation of the methods. The participants described experiences of deep emotional insight, self-reflection, and self-recognition, through engagement with the multimodal activities. The findings revealed that these approaches were: (1) credible, producing internally valid and contextually rich data; (2) contributory, generating original and applicable insights into family life; (3) communicable, offering accessible and structured ways for diverse participants to express their experiences; and (4) conforming, ensuring ethical engagement through inclusive participation. These findings demonstrate the potential of creative, arts-based, and participatory approaches, to advance methodological innovation in qualitative family research.

## 1. Introduction

Vulnerable and marginalised populations have consistently gained attention in research [1,2], however, this does not necessarily imply that they are being understood or heard. Due to research, a voice is provided for those who are under-represented, however, in order to do so, the research should be designed in a way that bridges marginalization and vulnerability [1]. Therefore, to ensure that the voices of vulnerable and marginalized individuals and communities are adequately represented, researchers should adapt their research design(s) to the contexts of the sample group(s) being explored [1]. This ultimately requires reconsidering every element of the research project, to ensure that the methodological tools and approaches are ethically sound, as well as contextually sensitive and suitable [2]. However, research has often been conducted on people from vulnerable groups, instead of with them [1]. Therefore, it is important to recognize vulnerable individuals and communities, not merely as statistics, but as active change agents in a participatory approach. This paves the way for researchers to think outside of the box, exploring alternative research methods that may provide deeper, more authentic insights [3]. Multi-modal methods support this shift, by creating opportunities for the participants to co-construct knowledge, share perspectives in ways that feel authentic to them, and contribute meaningfully to the research process [4,5].

In the context of this current study, we employed a range of multimodal research methods with families from two low-income communities situated in the Western Cape province of South Africa. These communities have been considered vulnerable, due to a multitude of socio-economic, environmental, and systemic challenges that cause their susceptibility to adversities [6,7]. Similarly, Nyathi et al. [8] highlight that the greatest disparities are experienced in rural areas, including high unemployment and poverty levels, which threaten social cohesion and inclusion. Families in these areas often face restricted access to essential support services, or employment opportunities, and are surrounded by prevalent social issues, such as substance abuse and crime [8]. According to an editorial article in The Lancet [9], such communities also have limited access to hospitals and safe water sources. Dlamini et al. [10] and Chinyakata et al. [11] concur, arguing that low-income parents face significant barriers in accessing healthcare, compared to their higher-income counterparts. Given the challenges faced by vulnerable families within these communities, the research approach was necessarily deliberate and purposeful. The use of multi-modal methods in this current study was not incidental, but intentional. By design, the choice of multi-modal methods for this research was aimed at generating authentic, participatory, and contextually grounded insights, which might otherwise have remained hidden, through conventional data collection techniques. Ultimately, multimodal methods were chosen to amplify the voices that are often silent, silenced, or unheard.

Importantly, participatory research carries implications that extend beyond the generation of knowledge. For instance, previous research suggested that to ensure the voices of marginalised and/or potentially vulnerable individuals are represented, researchers must adapt their research approach to suit the group being investigated [1]. Central to participatory approaches is the ethical commitment that research findings should be used to inform practices, programmes, and interventions that have the potential to improve the lives of the participant group, as well as the wider communities to which they belong. By engaging participants as active contributors rather than passive subjects, participatory research enhances the relevance, applicability, and contextual grounding of findings, increasing the likelihood that they can be translated into meaningful change. Previous studies have indicated that participatory and creative approaches support participants in being heard and represented on their own terms [4,5]. By valuing lived experience, storytelling, and co-created meaning, such approaches create spaces for individuals and families to share their realities in ways that reflect their priorities and contexts [4,5]. While recognizing that structural change often extends beyond the scope of a single study, participatory research can contribute to increased visibility of marginalised voices, inform advocacy and service design, and support processes through which communities may help to enact change over time.

The term, multimodal, may be described as any combination of qualitative and/or quantitative empirical methods of data collection, or analysis [12]. Multimodal research methods are uniquely suited to explore the experiences of vulnerable families in South Africa. These communities often face intersecting forms of marginalization, including poverty, exclusion from services, and historical trauma [8,13], therefore, capturing their realities through traditional verbal-centric research, risks overlooking important dimensions of their lives [14]. Multimodal strategies, such as LEGO^®^ Serious Play, drawings, photo elicitation, and meaningful objects, enable a deeper, more nuanced engagement with lived experiences that traditional qualitative approaches may not generate [15,16].

Traditional qualitative methods, such as interviews, focus groups, and ethnographies, have provided deep insights into understanding family dynamics, for many years. However, they often rely heavily on verbal or textual data, which could limit their capacity to capture nonverbal cues and embodied experiences [17,18]. This reliance may obscure crucial elements, such as gesture, eye contact, tone, and body language, all of which contribute to meaning-making in social contexts. Craig and colleagues [19] assert that this could reduce the interpretive richness and cultural sensitivity of findings. However, multimodal research strategies address these limitations, by integrating textual, visual, gestural, spatial, and auditory data [20]. These creative methods are especially beneficial for participants who struggle with verbal expression, children, trauma survivors, or individuals with limited education [21].

In addition, multimodal techniques offer participants a more active role in shaping research outcomes [22,23]. They reveal layers of family life, routines, relationships, resource allocation, and emotional dynamics, which interviews alone fail to capture [24]. For example, paintings and images allow the participants to express the domesticity of a place, as well as the materiality of space. Additionally, in family research, multimodal approaches enable exploration of how care, authority, or emotional labor, is communicated, not only through words, but also through creative acts, such as drawing or object elicitation. These techniques advance beyond rigid data collection structures, and offer participatory, as well as culturally attuned frameworks that promote inclusion and fairness [25,26]. Such methods are particularly beneficial for vulnerable families, where language, literacy, and age differences could affect communication. For instance, using visual tools, such as paintings, photography, and LEGO^®^, allows children, or less literate family members, to express emotions and memories that might be difficult to articulate verbally [21].

Multimodal methods enable storytelling, emotional engagement, and memory retrieval, in ways that traditional methods cannot [27,28]. By incorporating different modalities, researchers could access richer, more authentic representations of lived realities [29,30]. Storytelling, poetry, and visual expression offer non-threatening avenues for the participants to share their experiences, particularly where trauma or emotional distress may be present [31,32]. In this way, multimodal research enhances the emotional depth, as well as the authenticity of the data, offering new insights into how families interpret and give meaning to their lives [4,5]. While multimodal approaches provide valuable benefits for research [29,30], their application must be grounded in care. Accordingly, we, as researchers, followed the ethics of care in their multimodal approach, by prioritizing respect, inclusivity, and sensitivity to the lived realities of the participants, ensuring that the methods used were ethical [33].

The Ethics of Care theory is a relational approach to ethics that emphasizes the importance of human connection, responsibility, and responsiveness in caring relationships [33]. It is rooted in the understanding of care as a practice, rather than merely a feeling [33]. Multimodal approaches are aligned with this theory’s commitment to contextual, empathetic, and inclusive research practices. They allow for more holistic and care-centered representations of family life, by incorporating visual, narrative, and participatory methods [34]. This framework is focused on five moral elements, which are associated with the phases of care, namely, caring about (attentiveness), caring for (responsibility), care giving (competence), care receiving (responsiveness) and caring with (reciprocity) [33].

In this current multimodal approach, we intentionally embodied the principles of the Ethics of Care throughout data collection, in order to really ‘hear’ what the participant was saying. Caring-for involved acknowledging the vulnerabilities and challenges faced by the participants, as well as the importance of creating a supportive research space. Caring-for meant taking responsibility for the designing of research activities, such as drawing, LEGO building, and photo elicitation, which were inclusive to diverse participants. Caregiving was demonstrated in how the researchers of the study facilitated these activities with empathy, patience, understanding, and flexibility, creating a safe space for the participants to speak, by reducing the power imbalance between researcher and participant. Care-receiving was reflected in the researchers’ attentiveness to the participants’ responses and feedback, actively listening to their stories and engaging with them in a humanistic and respectful manner. Finally, caring with emphasized collaboration and co-construction of knowledge, where the researchers connected with the participants, built rapport, and treated them as partners in the research process, instead of passive subjects. By integrating multimodal methods within an Ethics of Care framework, we offer a relational, respectful, and context-sensitive approach to the understanding of families in vulnerable communities. It not only enhances methodological inclusivity, but also affirms the value and dignity of the participants’ experiences and expressions. Ultimately, they echo the core principles of the Ethics of Care: empathy, relationality, and responsiveness to individual needs [35,36]. While these approaches uphold ethical principles and methodological inclusivity, research on their use in exploring the experiences of vulnerable families remains limited, especially within the South African context.

Scant research has focused on the application of multimodal methods to understand families, particularly vulnerable families within the South African context [37,38]. A notable gap in the literature is the underutilization of innovative and participatory methods, such as draw-and-tell techniques, object elicitation, photo elicitation, I-poems, and other participatory tools, in the study of families. Previous research has revealed a need for innovative methods and strategies of data collection and generation, reflexivity and knowledge translation, to make qualitative research methods more inclusive [3]. Previously, those who communicated differently to the status quo, have been excluded from, or spoken for, in qualitative research [3]. The omission of these insights in the literature, therefore, constitutes a critical research gap [3]. This methodological gap represents missed opportunities to capture the nuanced, lived experiences of families through diverse and context-sensitive approaches.

This study addresses this gap by contributing to the growing body of literature on multimodal research approaches in South Africa. By adopting a multimodal approach, we introduce methodological innovations that transcend traditional forms of data collection, and offer multidimensional insights. Additionally, in this study, a participatory stance is embraced, engaging families directly in the research process. Therefore, this study aimed to explore the use of creative multimodal methods, within a Participatory Action Research (PAR) framework, grounded in care among vulnerable South African families [39].

## 2. Materials and Methods: A Careful Methodology

### 2.1. Study Design

This was a deductive, qualitative study, utilising a Human-Centered Design (HCD) approach [40], through Participatory Action Research (PAR) [39], to engage practitioners, families, as well as adolescents, to illustrate what families do and what their lived realities entail. The ultimate aim was to understand and strengthen families [41]. PAR is an approach to enquiry, which requires researchers to work closely with participants, to co-produce an understanding of a situation, to inform processes of positive social change, and to address inequality. Every family has its unique members, history, obligations, and customs. To understand “’doing’ family, it was essential to explicate and understand social life as it is enacted by real people in real situations” [42] (p. 34). Working with families and practitioners allowed for the exploration of how family was understood and described, through the generation and analysis of rich data. Similarly, to understand interventions, the relation between the abstract world of the family governed by others, and the concrete world of everyday family processes, rituals, events, and experiences, had to be clarified. PAR involved an iterative process that enabled consideration of the entwined relationship between what families ‘are’, and what families do.

**Figure 1:**
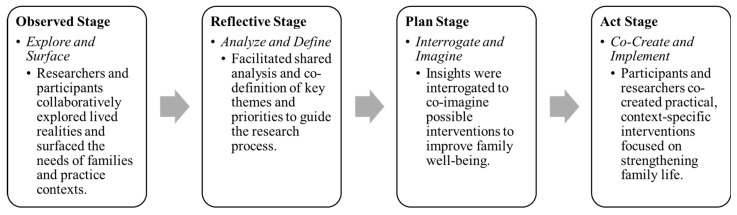
Steps in the Human Centered Design process.

Guided by the HCD approach, the researchers of this current study placed the experiences, needs, and capabilities of families, practitioners, as well as adolescents at the center of the research process. The PAR approach served as both a methodological and relational tool to support this design, enabling the participants to play an active role in shaping the research. PAR is aligned with the iterative nature of HCD, by incorporating four key stages, namely, observe, reflect, plan, and act. In the observed stage, the researchers and the participants collaboratively explored and surfaced the lived realities, as well as needs of families and practice contexts. The reflective stage facilitated shared analysis, as well as the co-definition of key themes and priorities, to guide the research. During the plan stage, insights were interrogated, to imagine possible interventions that could enhance family well-being. Finally, in the act stage, the participants and the researchers co-created practical, contextually relevant interventions, aimed at what could be changed to strengthen family life (Figure 1).

### 2.2. Location

Participatory Action Research (PAR) [39] was utilized, while working with families and practitioners in urban and rural communities supported by Cape Town and Saldanha Bay municipal governments. These communities were chosen because they were sites of enduring disadvantage and municipal government concern. Mfesane (a non-profit company), an independent, ecumenical development organization, has had longstanding experience in strengthening the capabilities of youths, as well as their families, who were experiencing significant disadvantages in both locations. Their embeddedness within the communities, as well as their daily relationships with young people and their families, were also important aspects of the management of risk within the project, namely, overcoming suspicion of outsiders, and maintaining long-term participation with young people and their families. Through Mfesane, family participants and various practitioners in the communities they served, were recruited to participate in the study.

### 2.3. Participants and Sampling

This research was conducted in partnership with families residing in two communities that were experiencing sustained socioeconomic disadvantage; one urban community located in Cape Town, and one rural community within the Saldanha Bay Municipality. A purposive sampling approach was employed, facilitated by the respective municipal governments, and supported by local agencies. This collaboration was essential, as these agencies held longstanding relationships with families in the communities, underpinned by trust, daily interactions, and social embeddedness. Participant recruitment combined purposive and snowball sampling techniques. Community workshops were hosted in familiar, accessible venues, such as a community hall, an early childhood development center, and a church. Initial access to families was mediated through family practitioners, who were themselves embedded in the communities. These practitioners also participated in the study, not in a professional capacity, but as members of the communities, with all data pertaining to their own families, instead of those they serve professionally. The total sample included participants from two locations: Saldanha Bay Municipality area (*n* = 70), and Mitchells Plain (*n* = 59). In the Saldanha Bay Municipality area, the participant distribution included 38 family practitioners, 16 parents, 5 adolescents, and 11 youths. The Mitchells Plain sample consisted of 12 family practitioners, 26 parents, and 21 adolescents. For validation purposes, an additional sample of 42 participants were recruited, including 9 academic staff members from the University of the Western Cape, 25 social workers based in Kimberley, and 8 postgraduate students (comprising both master’s and doctoral candidates).

### 2.4. Data Collection Procedure

**Figure 2:**
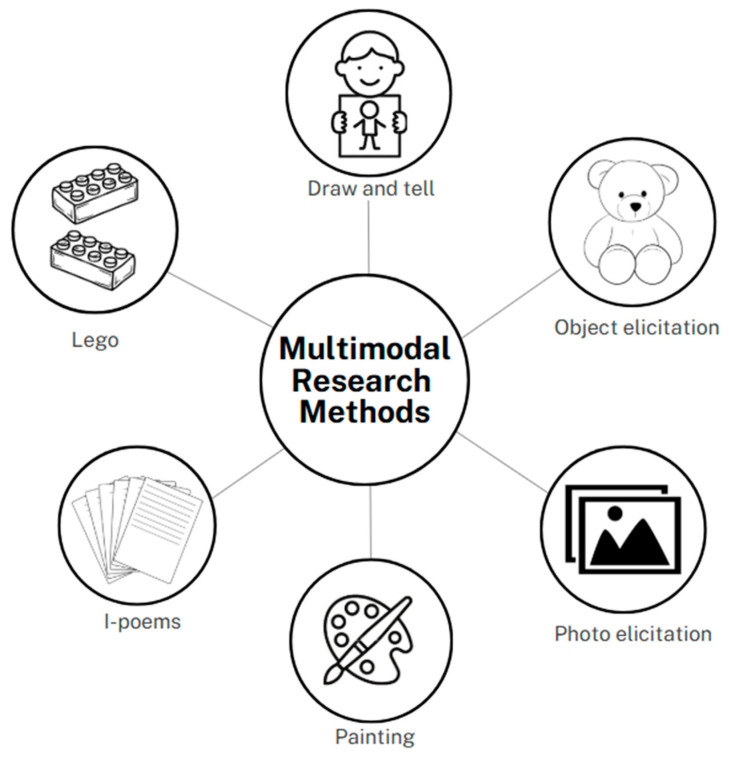
Multimodal methods employed to understand family capabilities.

Data was collected between July 2024 and April 2025 among parents, adolescents, youth and practitioners (social workers, as well as child and youth care workers) in community halls and churches. To gain a holistic understanding, a range of multimodal approaches were employed, in the form of participatory workshops, across three days. These methods helped build a foundational understanding of the culture of families, as well as what families do in their everyday experiences. This current study incorporated creative multimodal, sensory, and arts-based methodologies, to capture diverse perspectives, and deepen insight into family life, from the viewpoint of all participants. Innovative and alternative methods, such as photo elicitation, LEGO^®^ workshops, draw-and-tell activities, object elicitation, painting, and the listening guide (I-poems), were used. These approaches not only enriched the data, but also provided an initial baseline for an understanding of how families are perceived and experienced. The creative and innovative methodologies employed in this study are discussed below (Figure 2).

#### 2.4.1. Draw-and-Tell

Using the Draw-and-Tell method, the participants engaged in the creation of drawings to communicate their perspectives, feelings, or emotions, through diagrammatic representations [43]. This approach originated in the United Kingdom, in 1989 [44]. Initially, the participants were asked to create an image, followed by a series of written questions. However, this was later replaced with the Draw-and-Tell technique, which integrates interview questions, facilitating a more interactive and reflective process [44]. The drawings serve as a medium for engagement between the participants and the interviewer, allowing data to be collected through reference to the drawings, as well as the participants’ accompanying narratives [45]. According to Goodwin et al. [44], the Draw-and-Tell technique involves the participants creating drawings, followed by discussions about those drawings, promoting a deeper exploration of perspectives and experiences. The focus is first placed on the participant’s drawing, and subsequently, the feelings, perspectives, and emotions embedded within the drawing are externalized into concrete form, which could be reconstructed and analyzed [46]. In this current study, the Draw-and-Tell method was chosen for its ability to create a relaxed and non-threatening environment, helping the participants feel at ease. Parents or caregivers, as well as practitioners were asked to draw a plan of their house, as seen from a rooftop view, while adolescent children were asked to draw representations of their families. The participants were provided with white paper and a variety of drawing instruments, such as pencils and crayons in various colors. This technique was particularly beneficial, as it fostered interaction and conversation around the drawings, allowing the participants to share, as well as discuss their ideas freely. The Draw-and-Tell process initiated meaningful discussions about families, enabling the participants to reflect on what family means and what families do. This stage was also instrumental in helping families to understand the research process, and be comfortable as active co-designers. The practitioners facilitated unstructured discussions, focusing on the process of creating the drawings, and the final representations of family life. These discussions were audio-recorded, while the drawings were annotated and collected for analysis, providing valuable insights into the participants’ perspectives and experiences of family life.

#### 2.4.2. Painting

Painting was employed as a multimodal method to explore the participants’ perceptions of their lived realities, communities, and the changes they envision within family life. This method allowed the participants to represent their experiences visually, particularly focusing on their family dynamics [45]. The participants were provided with paint, paintbrushes, and paper to create their artwork. Once completed, the paintings served as a focal point for reflection, during which the participants discussed the colors that they had selected, as well as how these elements symbolized various aspects of their reality. This reflective process fostered a deeper connection to the themes of family capabilities, allowing the participants to express complex emotions and ideas that may not have been easily conveyed solely through words. Following the creation of the artworks, the participants’ reflections were captured and integrated into the analysis, providing valuable insights into their personal and collective experiences. Subsequently, the artworks were annotated, combining the visual representations with the participants’ interpretations, for a more comprehensive understanding of their lived experiences.

#### 2.4.3. Object Elicitation

Object elicitation is a qualitative research method that employs objects, for example, photographs, artifacts, or personal belongings, as prompts for discussion, reflection, and meaning making [47,48]. This versatile methodology engages participants, by utilizing tangible objects that hold personal significance, enhancing qualitative research [26]. According to Willig [49], object elicitation was designed to facilitate the participants’ communication regarding aspects of their experiences that may have been difficult to articulate solely through words. O’Brien and Charura [50] conceptualized object elicitation as a decolonizing approach to research, aiming to dismantle systems of power disparities. The participants were invited to bring along objects of personal significance, with the objects chosen being relevant to the study, as well as reflective of the participants’ everyday lives and experiences.

In this study, the participants were asked to bring along objects to the workshop that held various personal significance. These objects, which included items, such as lipstick, earrings, books, Bibles, walking sticks, lighters, and dominoes, were used to elicit responses and encourage reflection. The personal significances contained in these objects helped to evoke emotions and memories of people or events, providing rich data on the participants’ lived experiences [47]. The process allowed the participants to reflect on their individual and collective identities, facilitating the exploration of the sensory and emotional dimensions of what families do. Family members were encouraged to select an object that represented their family, and invited to discuss its relevance, as well as meaning within the context of their family setting. This approach enabled a deeper understanding of the multimodal nature of family experiences, particularly the role of materiality and emotion in constructing familial identities. Data from these discussions were audio-recorded and transcribed for analysis, providing valuable insights into how objects serve as metaphors for family dynamics and relationships.

#### 2.4.4. Photo Elicitation

Photo elicitation is a qualitative data collection method that uses photographs, or other visuals, as prompts in interviews, to encourage deeper reflection, memory recall, and discussion [51,52,53,54]. Originating from visual anthropology and sociology, photo elicitation involves engaging with pre-existing images, allowing the research participants to reflect on them during the interviews [52]. Willig [49] highlighted that photo elicitation facilitated the participants’ expression of their opinions, values, beliefs, and experiences, with photographs serving as tools to stimulate dialogue and support personal reflection. This method enhances traditional verbal interviews, by introducing visual stimuli that could evoke emotions, trigger memories, and promote storytelling [55].

In this research, photo elicitation was employed to provide the participants with the opportunity to speak freely, while the researcher stays neutral. According to Poku [56], photo elicitation helps to address power dynamics, encouraging active participation and engagement, particularly in family-focused research. The approach fostered interaction, alleviated anxieties, and helped build rapport between the researcher and the participants, allowing them to feel at ease to share their experiences [51]. By using photographs, the participants were able to articulate their thoughts, experiences, and emotions more freely, generating rich, in-depth data [53]. Unlike traditional verbal interviews, photo elicitation interviews extract a broader range of information, often resulting in longer, more detailed discussions [51,52].

The participants in this current study brought photographs that were personally significant, including pictures of themselves, their family, extended family members, a place, a landscape, and even a tree. These images were chosen for their emotional and personal relevance. Family members were encouraged to take photographs in their home environments and beyond, illustrating what it meant to be a family, as well as the activities they shared. This allowed the participants to have agency in how they represented their family life [57]. The goal was for the photographs to capture, not just static moments, but also the dynamic nature of family life, including action and movement. Subsequently, the practitioners discussed the photographs, exploring the intentions behind each image, and how they represented what families do. This process created visual displays, with annotations that reflected the activities in which families engaged, together and separately, while also capturing the voices of the family members [58]. The data from these discussions were audio-recorded and transcribed for analysis, offering rich insights into the ways in which families construct and experience their identities.

#### 2.4.5. I-Poems

The Listening Guide (LG) is a qualitative data collection and analysis tool, primarily used to explore the deep, interpretive analysis of spoken, or written narratives. It focuses on capturing the complexities of individual experiences, identity, and voice. Woodcock [59] describes the LG as a relational, voice-centered, feminist methodology that is particularly useful in analyzing interview transcripts. This methodology places significant emphasis on the importance of voice, particularly through the creation of ‘I-poems,’ which are used to capture the subjective experiences of the participants, in their own words. The LG involves a structured approach that incorporates multiple listens to data, leading to a deeper understanding of the participants’ perspectives, as well as the sociopolitical contexts that influence their narratives. The process consists of four steps: (1) listening for the plot; (2) listening for the ‘I’ voice; (3) listening for contrapuntal voices; and (4) listening for broader political, social, and cultural structures [59,60]. In the second step, listening for the ‘I’, researchers focus on the use of the first-person voice, capturing each ‘I’ statement, and organizing them into I-poems. This method allows for a deep exploration of how individuals express their sense of self, with each ‘I’ statement reflecting a distinct perspective or emotion [61]. Edwards and Weller [62] further highlighted the utility of I-poems in capturing the subjective experiences of family members. In their research, they applied the LG to analyze family narratives, particularly focusing on the personal subjective experiences of what families do. After transcribing the data from the discussions, they created I-poems, by identifying instances of ‘I’ expressions within the transcripts. Subsequently, these poems were shared with family members, to encourage reflection on their own roles and experiences within the family. This process facilitated a deeper engagement with the subjective expressions of family members, linking back to the focus on activity and interaction within families. The process was innovative, engaging families in reflection on their subjective experiences and roles, while contributing rich data about family dynamics, and their sense of belonging.

In this study, the LG was applied to explore the narratives of adolescents, their parents, and practitioners, within families from resource-constrained communities. The iterative reading of transcripts allowed for the extraction of voices, in the form of I-poems, amplifying the perspectives of marginalized groups, and enhancing the representation of their voices. This approach facilitated a detailed exploration of how family members articulate their identities, relationships, and experiences, contributing to the understanding of family dynamics, in contexts of limited resources. Through this method, the study gained a nuanced understanding of the individual and collective expressions of families, highlighting the emotional and relational aspects of their experiences.

#### 2.4.6. LEGO^®^

LEGO^®^ Serious Play^®^ (LSP) is one of the methodologies utilized in this current study to facilitate deeper engagement and understanding. As an innovative facilitation methodology, LSP uses LEGO^®^ bricks to assist the participants to articulate their ideas and concepts, through concrete representations [63]. Developed in the 1990s and influenced by psychological theories of learning, play, and constructionism, LSP allows the participants to think with their hands, building symbolic models that represent their emotions, thoughts, and experiences [64]. This approach is designed to move qualitative research beyond traditional word- and text-based methods, advancing the exploration of family dynamics through creative, tactile engagement [65]. The LSP methodology was implemented in workshops with families and practitioners, where the participants engaged in a three-step process; building, sharing, and listening. Each participant created a tangible LEGO^®^ model to represent his/her perspective, which subsequently, was shared with the group, fostering communication, collaboration, and mutual understanding [64]. This hands-on approach not only allowed the participants to reflect on their experiences, but also enabled them to actively engage with the ideas of others [66]. Observations from the workshops revealed that the LSP approach helped to level the power dynamics between the participants and the researcher, creating an egalitarian space, where all voices were equally valued. LEGO^®^-based methods served as an inclusive tool to capture the knowledge and perspectives of the participants, ensuring that marginalized voices were heard. The methodology aligned with qualitative research by incorporating storytelling, narrative construction, and visual communication, enabling the participants to express their thoughts in a non-evaluative environment. This process facilitated the co-creation of knowledge, allowing research to capture present experiences, as well as speculative futures, transcending traditional social science methodologies. In this current study, LEGO^®^ was not merely a data collection tool, but also a catalyst for the exploration of complexity, generating actionable insights, and transforming qualitative research practices.

The instructions to the participants regarding the workshop activity as well as examples of the types of questions and probe questions that were asked during the sessions, are provided in Table 1. Since this current study was deductive in nature, these questions were interpretive and unstructured, based on the responses and narratives provided by the participants; however, also bearing in mind the theory of the Ethics of Care, theorized by Tronto [33]. The participants were instructed that they had full autonomy to decide whether they would like to engage with the questions or not.

### 2.5. Data Analysis

The audio-recordings were transcribed verbatim in English and uploaded to Atlas Ti. Version 8 for coding, and thematic analysis was conducted across all data forms [67]. Thematic analysis was conducted, commencing with the familiarization with the data, during which transcripts, visual artefacts, and creative outputs were repeatedly read, viewed, and reflected on, to gain a comprehensive understanding of the participants’ expressions. Initial coding was undertaken next, with verbal, as well as visual data coded manually, to capture descriptive and interpretative meanings (for example, quotes linked to family communication were coded as FC_ Participant 1_Mother_Vredenburg, or FC_ Participant 2_ Father_ Mitchells Plain). Textual data were coded for recurring ideas, emotions, and language patterns, while visual data, such as drawings, paintings, photographs, and LEGO constructions, were explored for color, form, symbolism, and spatial arrangement. Related codes were subsequently grouped into potential themes, which were reviewed and refined through iterative comparison, across data sources, to ensure coherence and analytical depth. Later, each theme was defined and named to reflect its central meaning, while maintaining closeness to the participants’ voices and visual representations. The process concluded with the development of an analytic narrative that integrated the visual and verbal dimensions of the data, illustrating how the participants conveyed meaning and experience, through multiple modes of expression.

#### 2.5.1. Draw-and-Tell

Draw-and-tell activities invited the participants to create visual depictions of family life and personal experiences. During analysis, the drawings were explored independently first, with attention given to color choice, spatial layout, and represented relationships. The accompanying verbal explanations were transcribed and analyzed next, for emotional tone, meaning, and interpretive commentary. Integration of the visual and verbal data provided insight into the symbolic, as well as the spoken aspects of the participants’ stories.

#### 2.5.2. Painting

Paintings offered a more abstract and expressive form of communication. These artworks were analyzed by observing the use of shapes, patterns, and imagery, to identify emotional or conceptual themes. Notes made during painting sessions, as well as the participants’ reflections on their artwork, were incorporated to understand the intentions and meanings behind the visual elements. This process enabled a deeper interpretation of how the participants represented emotions and relationships through visual art.

#### 2.5.3. Object Elicitation

During object elicitation, the participants brought, or selected objects (such as a hairbrush or seashell) that represented aspects of family life, or personal meaning. Each object was discussed in an unstructured conversation, and these narratives were transcribed for analysis. Attention was paid to the descriptions, memories, and associations linked to each object. The visual and symbolic qualities of the objects were also noted, to capture the emotional and contextual dimensions of participants’ interpretations.

#### 2.5.4. Photo Elicitation

In the photo elicitation process, the participants brought along photographs that reflected their everyday experiences of family life. The photographs were explored for visual content, composition, and contextual symbolism. The participants’ discussions of their chosen images were analyzed, to understand how they interpreted and articulated the meanings behind their photographs, in terms of space and place (such as a household farm). This dual analysis allowed the research team to connect visual imagery with the participants’ personal narratives and reflections.

#### 2.5.5. I-Poems

I-poems were developed as part of the LG approach. The transcripts were analyzed, using the Listening Guide approach [60]. This approach involved multiple readings of the data, to trace the participants’ voices, emotional tones, and narrative shifts. The LG was appropriate for the expressive and layered data, generated through creative methods, as it allowed for an in-depth understanding of how the participants made meaning of family life and capabilities, within their specific contexts. The analysis was guided by four steps: (1) listening for the plot to identify overarching storylines and emotional patterns; (2) constructing I-poems to trace the participants’ self-representations and inner experiences; (3) listening for multiple or conflicting voices within individual narratives; and (4) composing a final analysis that synthesized insights, while remaining close to the participants’ voices and expressions. The I-poems were analyzed to explore how the participants represented themselves, their emotions, and their sense of identity. This technique provided a direct way of hearing the participants’ inner voices, as well as capturing subtle shifts in tone and perspective.

#### 2.5.6. LEGO

LEGO-based activities encouraged the participants to physically build representations of family life and relationships. The constructions were observed for structure, color, and symbolic elements, while the participants explained their models during follow-up discussions. These verbal accounts were transcribed and analyzed, to identify how physical building choices related to the participants’ conceptualizations of family dynamics. The combination of visual observation and narrative interpretation provided a tangible view of the participants’ experiences and values.

The multimodal approaches were integrated to enable a rich and comprehensive understanding of the data. Each method contributed a unique perspective, and together they provided a holistic view of how the participants articulated and visualized their experiences of family life and dynamics.

### 2.6. Trustworthiness

The concept of trustworthiness in qualitative research refers to the extent to which the findings are accurate and meaningful, from the perspectives of the researcher, the participants, and the reader [68]. In this current study, trustworthiness was grounded in an ethic of care [33], which prioritized empathy, mutual respect, and attentiveness to the lived experiences of families. Guided by this ethic, PAR was conducted, in close collaboration with family members, to co-create knowledge and ensure that their voices were authentically heard. An ethic of care was operationalized through sustained engagement, active listening, and responsiveness to the participants’ needs and insights, across the research process. This collaborative and caring approach enhanced credibility, as interpretations were validated through ongoing member or stakeholder checking. To enhance credibility, participants were actively involved in validation processes. This included reviewing and discussing preliminary findings and themes, providing feedback on visual artefacts (for instance, I-poem books, drawings, LEGO^®^ constructions, photos), and confirming whether interpretations reflected their experiences accurately. Some participants were invited to co-interpret multimodal outputs during workshops, while others provided written or verbal feedback on summaries of the findings. These processes enabled participants to correct misinterpretations, clarify meanings, and suggest refinements to the findings. For example, the I-Poem books were provided to participants to allow them to see their lives represented in poetic form and to reflect on their lived realities. This was followed by discussions with each participant about what their poem meant to them and how it reflected their personal experiences and perspectives. Rich, thick descriptions were used to present the findings in a way that allowed readers to develop a nuanced understanding of the participants’ realities [68]. In addition, dependability and confirmability were supported through transparent documentation of the research procedures and decisions, reinforcing commitment to relational accountability and ethical responsibility.

### 2.7. Ethical Considerations

Ethics approval and permission from the university were obtained prior to the commencement of this current study, reference number: HS24/3/28. All stakeholders, who voluntarily participated in all methods in this study, provided their consent and signed a confidentiality binding form. Before the start of the data collection session, the participants were reminded that their participation in the study was voluntary, and that they could stop and withdraw at any stage, without any penalty. To ensure confidentiality and anonymity, the participants’ names were removed and replaced with pseudonyms.

## 3. Results

A range of creative arts-based multimodal approaches were employed in this current study, to explore the lived experiences of South African families, practitioners, and adolescents, and understand the notion of family capabilities. These creative participatory approaches were employed deliberately, to engage with the senses of sight, sound and touch. This provided the participants with the opportunity to express and communicate their familial experiences. For some, for example, adolescents, traditional methods of qualitative research, such as interviews and focus group discussions, were restrictive and intimidating. In contrast, methods like LEGO^®^ and painting provided a creative outlet for individuals to express themselves when they did not have the words to speak. This ultimately highlights the importance of utilizing multimodal approaches, to provide a space for the participants to have their voices heard. To ascertain the robustness and relevance of these multimodal methods, the following table (Table 2) is presented, according to the conceptual hierarchy of research quality [69]. This framework includes four interrelated quality dimensions: credible, contributory, communicable, and conforming. Table 2 demonstrates how each multimodal method met these quality criteria, in the context of this current study, and emphasize their unique contribution in understanding the complex nature of families through quotes.

### 3.1. Draw-and-Tell

Participants used drawings to represent their households, family members, and the spaces each occupied, allowing them to articulate family life visually (Parents’ depiction of household) These drawings provided credible insight into household structure, roles, and relational dynamics, while participants reflected on and discussed their creations with researchers and peers. One parent noted that drawing their home helped them “see how everyone fits in and relates to one another,” highlighting how the method supported understanding and validation of their lived experiences. Drawings also contributed to knowledge creation by revealing symbolic expressions of care, responsibility, and belonging. The visuals were highly communicable, allowing participants to share their realities with others in the group and with researchers, fostering discussion and mutual recognition. Importantly, participants retained control over the content and scope of their drawings, ensuring ethical participation and autonomy, particularly for adolescents who were able to engage on their own terms.

### 3.2. Photo Elicitation

Through sharing personal photographs of family gatherings, recreational activities, and special occasions, participants grounded discussions in real-life experiences of space and place (Photo of a family). These photos enhanced credibility by anchoring reflections in tangible moments and spaces, while prompting participants to verbalize the significance of each image. One participant described the activity as “very thought-provoking… it makes you talk about your life,” highlighting the method’s capacity to generate authentic narratives. The photos were contributory, revealing everyday routines, traditions, and relational dynamics, and offered insight into culture, memory, and identity. They were also communicable, enabling participants to explain experiences visually and verbally to fellow research participants and researchers. Participants maintained agency and ethical engagement by choosing which photos to share and the depth to which they discussed their family stories, reinforcing respectful participation.

### 3.3. Object Elicitation

Meaningful objects, such as a Bible, hairbrush, seashell or medal, prompted participants to reflect on familial dynamics in a tangible way (Objects used to relate to family dynamics). Credibility was enhanced as these objects anchored discussions in lived realities, with participants sharing the emotional and symbolic significance of each item. The approach was contributory by uncovering values, family priorities, and relational tensions, providing culturally and contextually rich insight into family life. Communicability was strengthened as abstract experiences were expressed concretely and memorably, facilitating discussion among participants and researchers. The process was conforming, as participants selected the objects themselves and shared stories at their own pace, promoting ethical engagement and reflective sharing.

### 3.4. Painting

Participants expressed emotional bonds, family unity, and relational challenges through paintings, with some depicting hands holding to symbolize support, while others illustrated broken households or changes they wished to see in family dynamics (Painting of family household relationships). The paintings enhanced credibility by providing a non-verbal, emotionally resonant representation of participants’ lives, while enabling discussion and reflection on the images. Contributory value emerged as the artworks offered insight into family functioning, communication, and coping mechanisms, particularly in contexts where emotional experiences are often unspoken. Communicability was high, as visual narratives coupled with participant explanations made complex experiences accessible to both peers and researchers. The method was conforming, with participants retaining control over content, depth, and sharing, allowing voluntary, self-directed engagement that promoted psychological safety.

### 3.5. LEGO^®^

Participants constructed 3D LEGO^®^ models of their households and shared spaces, verbally explaining roles, relationships, and power dynamics (LEGO® interpretation of familial dynamics). This enabled participants to ‘think with their hands’. Credibility was reinforced as the models’ helped participants externalize abstract family dynamics and reflect on personal experiences. For instance, one participant remarked, “This LEGO^®^ made me realize something I hadn’t acknowledged before.” The approach was contributory by highlighting tensions, aspirations, and coping mechanisms, with recurring themes such as love, respect, and conflict observed across multiple models. Communicability was strong, as the models visually and verbally conveyed family structures to peers, researchers, and community practitioners, fostering discussion and mutual recognition of experiences. Participants retained agency and autonomy in building and sharing their models, allowing for a safe, playful, and ethical environment for ‘hands-on’ expression.

### 3.6. I-Poems

I-Poems, constructed directly from interview transcripts, preserved participants’ voice, cadence, and emotion, enabling them to see their lives represented in poetic form (Adolescent I-poem). This generated credibility as participants reflected on their own experiences and validated interpretations. For example, one mother commented, “Wow… it’s like I saw myself in this poem… the life I’m living for my family,” illustrating deep engagement and reflection. Contributory insights emerged as poems revealed emotional layers such as hope, responsibility, care, and fear, which might not have been captured through traditional interviews alone. The poems were communicable, shared among participants to stimulate discussion and recognition of common realities. Conforming was ensured as participants reviewed their poems, discussed meanings, and provided consent for use, maintaining ownership of their narrative.

## 4. Discussion

In this current study, we explored the use of creative multimodal methods, within a participatory action research framework [39], grounded in care among vulnerable communities. Families are frequently faced with intricate emotional dynamics that might be challenging to convey in traditional research settings [14]. Therefore, to reach this aim, multimodal methods were employed, namely, Draw-and-Tell, photo and object elicitation, painting, LEGO^®^, and I-poems. These methods provided innovative and alternative approaches to understand family dynamics, which moved beyond traditional methods, as they allowed the participants to express their experiences and viewpoints, without using only spoken or written language [57]. However, to ensure that these multimodal methods could be replicated by other researchers, it is important to discuss their research quality, specifically their credibility, contributory value, communicability, and conformity.

### 4.1. Credibility

The multimodal approaches employed in this current study were found to be credible, due to their rigorous, contextual and internally valid design. Each method allowed for the elicitation of consistent and coherent narratives that reflected the lived realities of family life [70,71]. For instance, object elicitation supported transparency in the meaning-making process, as the participants chose personal items to represent their family experiences [72]. This ensured that the interpretations remained grounded in the participants’ own context. Similarly, photo elicitation revealed reliable patterns in emotional responses, helping to verify relational themes that were echoed across these multimodalities [73]. LEGO^®^ construction offered a unique, but repeatable structure through which the participants were able to articulate their family roles and responsibilities, often in metaphorical, yet internally consistent ways. The Draw-and-Tell method, as well as painting, enabled the participants (especially the adolescents or less verbal individuals) to share complex emotions with clarity, by capturing individual voices in a structured and transparent manner. These poetic representations, not only of themselves, but also of their family dynamics, maintained internal coherence, and preserved the participants’ self-expressions with fidelity. Additionally, it facilitated the engagement of multiple senses, to facilitate the expression of experiences, allowing the participants to externalize emotions, relationships and memories [73]. This ultimately contributed to the overall credibility of the subsequent findings.

### 4.2. Contributory

Each multimodal method contributed original procedures and relevant findings that extend existing knowledge on family capabilities. For example, the object, as well as photo elicitation generated original ideas, through the participants’ selection and interpretation of everyday images and items. This resulting in applicable insights into how families express care and support. The LEGO^®^ activity, as a participatory method, introduced a tactile, constructivist dimension that is still underused and understudied in family research. Therefore, its adaptability and expressive nature render this method current and innovative. Aligned with a previous study, drawings may elucidate issues, such as exposure to violence, feelings of loneliness or exclusion, and the necessity for attention and support [74]. In addition, the painting and Draw-and-Tell approaches uncovered emotional undercurrents in family dynamics, which ultimately produced visually rich data that added depth to thematic interpretations [75]. Notably, the construction of I-poems from the Listening Guide approach contributed novel results, by foregrounding the ‘I’ narrative. This emphasized the role of individual voices in the shaping of collective family realities, and consequently, a deeper understanding of family capabilities could be depicted. Ultimately, this facilitates creative thinking and problem-solving [63], which, in turn, strengthens positive identity formation and self-efficacy, so vital to family well-being [76]. Together, these methods produced findings that were contextually relevant, and potentially generalizable to broader contexts concerned with family relational dynamics.

### 4.3. Communicable

A key strength of this current study was the accessibility and readability of its findings, made possible through the inherently structured and consumable nature of the creative outputs. The object and photo elicitation methods generated materials that could be visually and thematically catalogued, consequently, making the data more searchable and engaging for varied audiences. The LEGO^®^ models, with their spatial logic and metaphorical richness, enabled the findings to be interpreted visually, as well as narratively. This allowed complex family structures to be more understandable by the researchers and stakeholders alike. These results align with previous research, where it was reported that art-based methods are effective, when working with children, as they allow children the opportunity to use their imaginations as a way of opening up and expressing themselves [73,77]. Drawings and paintings, as visual artefacts, were highly communicative in this current study, as these offered direct emotional resonances, and prompted dialogue among the research team and the participants. More specifically, the I-poems, presented in a poetic form, transformed raw transcripts into structured, readable narratives that conveyed emotional intensity and identity, in a condensed, powerful manner. These methods shift the power dynamic, enabling the participants to become co-designers of information, instead of mere contributors [78]. This is especially important when working with families, as it fosters a sense of agency and collaboration, which ensures that their perspectives are reflected accurately in the research findings [57]. Families are more likely to recognize their own abilities and strengths when they take an active role in deciding how their experiences are documented and interpreted [57]. Similar to previous authors, the methodological approaches created flexible, participant-driven spaces that provided individuals with the opportunity to express their lived experiences [71,79]. Collectively, these approaches strengthened the communicability of the research by inviting diverse forms of engagement. Additionally, in this current study, we demonstrate that the multimodal approach facilitated the articulation of emotions, and fostered an engaging environment conducive to the expression of feelings [63].

### 4.4. Conforming

The utilization of creative multimodal methods is aligned with ethical, morally justifiable, and inclusive research practices. Each approach promoted equal opportunities for participation, by providing individuals with the opportunity to choose their preferred method of engagement, which suited their comfort, language abilities, and personal expression. The study was conducted in resource-constrained communities; therefore, the multimodal approach facilitated the capture of complex life experiences and sensitive context [19]. Consequently, arts-based research methods provide social scientists with a diverse array of techniques to engage participants, who are frequently marginalized, in more conventional research paradigms [70]. For example, Draw-and-Tell, as well as painting, were particularly inclusive for the adolescents, or participants, who found verbal interviews challenging, while the I-poems allowed space for validation, reflection, and personal agency to voice expressions. Similarly, Cetin and Gunes [80] found that when utilized in research involving children, drawings provided researchers with valuable insights into themes, such as love, communication, and daily activities, among others. These methods were also conducted in ways that were open and transparent, with the participants actively involved in the interpretation and validation of the data [80]. Importantly, the employment of multi-model methods adhered to all relevant ethical regulations, ensuring that participation was voluntary, confidential, and emotionally safe, as well as comfortable. The emphasis on co-constructing meaning fostered a sense of shared ownership over the data, which ultimately contributed to sustainable and equitable research practices that respected the participants’ dignity and contribution.

### 4.5. Strengths and Weaknesses

The strength of this current study lies in its methodological innovation. Traditional research approaches could frequently portray the participants as inactive subjects, instead of engaged contributors to knowledge creation [81,82]. In contrast, multimodal approaches provided the participants with the chance to take ownership of their stories [83,84]. By employing a range of creative, arts-based multimodal methods, such as draw-and-tell, photo and object elicitation, painting, LEGO^®^, and I-poems, we created flexible, sensory-rich spaces for the participants to articulate family life in their own terms. This approach was particularly valuable in engaging children, adolescents, and participants who might have struggled with verbal expression in traditional interview settings. The combination of methods enhanced the credibility and communicability of the data, as reflected in the diverse, emotionally resonant, and contextually grounded insights that emerged. Another strength was the participatory action ethos underpinning the research. The practitioners, who were embedded in the communities, contributed as gatekeepers, as well as participants, which enriched the depth of data, and supported ethical and culturally respectful engagement. The alignment of the methods with the hierarchy of research quality [69], further underscored the robustness of the approach, in terms of credibility, contribution, communicability, and conformity.

A key strength of this study was the researchers’ reflexive awareness of the potential for power imbalances and social desirability within participatory research contexts. Rather than assuming these dynamics could be removed, the research design acknowledged that participants might respond in ways they perceived as aligning with researcher expectations. This awareness informed the intentional use of multiple multimodal, creative methods, which offered participants varied and flexible ways to express their experiences beyond researcher-led verbal questioning. The availability of different modes of engagement allowed participants to select approaches that felt most comfortable, thereby supporting more participant-led meaning-making. At the same time, these dynamics remain a methodological consideration. Despite reflexive practices and the use of multimodal methods, power relations between researchers and participants may still have influenced participation and disclosure, particularly given the involvement of vulnerable families. Engagement across methods varied, and some participants may have participated selectively or shaped their responses accordingly. The inclusion of a validation phase helped to address this by allowing participants to confirm or challenge interpretations, however, these dynamics cannot be fully eliminated. Acknowledging them enhances the transparency and trustworthiness of the study and provides important context for interpreting the findings.

Nevertheless, some limitations existed, which needed to be acknowledged. Although the multimodal approaches provided inclusive avenues for participation, not all the participants engaged equally across all methods. For instance, some may have skipped the question and probe rounds in the LEGO^®^ workshops. Preferences and levels of comfort varied, which may have influenced the type and depth of the data collected. Some of the participants, who were more artistically inclined, found joy in the painting activity, while others thought that their creativity was not on par with the rest of the group. Additionally, the reliance on purposive and snowball sampling, while appropriate for family-based research, may have limited the generalizability of the findings beyond the two study sites in Cape Town and the Saldanha Bay Municipality. The interpretive nature of visual and creative outputs also presented challenges in analysis, particularly related to researcher bias and over-interpretation. While reflexivity was maintained throughout, the subjective dimension of interpreting drawings, paintings, and LEGO^®^ models remained a methodological consideration.

### 4.6. Future Research

In terms of future studies, researchers wishing to conduct similar research initiatives may consider and adopt longitudinal designs, to track changes over time. This would enable a more robust and comprehensive analysis of: (1) changes in family behaviour and dynamics; (2) the use and validation of creative arts-based methodologies; and (3) strengthening the understanding of family capabilities. In addition, future researchers could provide the participants with the option of choosing their preferred method of creative arts-based engagement. This would provide a space in which the participants would feel comfortable and safe to share their deepest thoughts. Additionally, broadening the study to different locations within South Africa may enhance the generalizability of the study, as this would improve the transferability of the results across different contexts and locations.

## 5. Conclusions

In this study, we explored the use of creative multimodal methods, within a participatory action research framework, grounded in care, among vulnerable communities. Additionally, we demonstrated how multimodal methods could serve as powerful, inclusive, and contextually sensitive tools, to explore family capabilities. By engaging the participants through their senses (touch, sight, and hearing), methods such as draw-and-tell, photo and object elicitation, painting, LEGO^®^, and I-poems, enabled the articulation of complex family experiences, which might have remained inaccessible through conventional qualitative research methods only. These approaches were particularly important in supporting the voices of children, adolescents, and community-based practitioners, many of whom face structural or expressive constraints, in more formal research settings. Framed within the hierarchy of research quality, the multimodal approaches demonstrated strong credibility, communicability, ethical alignment, and meaningful contribution, to theory, as well as practice. In this current study, we highlighted the potential of creative, arts-based, participatory methods to advance methodological innovation in family research.

## Figures and Tables

**Table 1:** Example of workshop instructions, questions and probe questions.

Multimodal Method	Instruction	Question	Probe Questions
Draw-and-Tell	Draw us your family household (your place and space).	Who occupies this place and space?	Who is in this house? What are the roles and relationships like in this place and space? Who takes responsibility?
Object elicitation	Bring an object that means something to you regarding your family.	Tell us about your object.	What does your object represent? What does your object mean to you?
Photo elicitation	Bring a photo that relates to family (it can be an image of your family, things, space and/or place).	Tell us your story and about your family.	Who are the people in this photograph? (What do they mean to you? What is your relationship like with them?) Was this a particular occasion? (Why did you celebrate this occasion?/How did you feel?)
Painting	Paint us a picture, starting from approximately 10 years old (as an adolescent) to where you are now (as an adult), of how your family has changed over time.	What does your family life look like?	What has changed from the time you were an adolescent to where you are now in your adult years? What changed the most for you? (Do you think this change has had a positive or negative impact on your life?) Is there anything you still want to change? (How can we make that change?) Any particular reason why you chose certain colors to depict your family?
I-poems	Read your I-poem and reflect on it for about 10 min.	Tell us what this poem means to you.	When you read this poem, how did you feel? (Why did you feel that way?) What parts of this poem stand out the most for you? Who takes care of you?
LEGO^®^	Build how you see yourself. Build how your family sees you.	How do you see yourself? How does your family see you?	Why do you see yourself in this manner? (What made you see yourself in this manner?) What does care look like in your family? Why do you think your family sees you in this manner? (Who in your family can you ask for help? Why can/can’t you ask that person for help?) Who is attentive to your needs?

**Table 2:** Research quality of multimodal methods.

Multimodal Method		Research Quality Component			
Methods	Visual Example from Empirical Data	Credible	Contributory	Communicable	Conforming
Draw-and-Tell	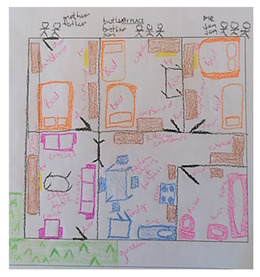 Parents’ depiction of household	Participants used drawings to represent family life, such as their household and the various sections that family members occupy. These visuals allowed not only us as researchers to understand their household, but also for other participants to relate to similar circumstances and to depict their story that was authentic and felt safe.	Revealed how families, practitioners, and children experience family life through symbolism and relational depictions, offering insight into real-life perspectives on structure, care, and belonging.	Highly engaging during dissemination; helped communicate complex ideas visually to both academics and practitioners, especially in the plenary feedback sessions. “I’ve tried to draw it because I can’t say a lot.” (Participant 1)	Provided autonomy over what was shared and respected participants’ agency in shaping the narrative. This method also allowed the adolescents to participate on their own terms.
Photo Elicitation	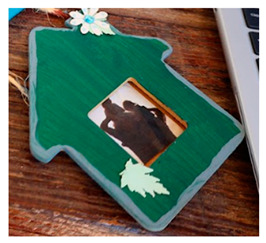 Photo of a family.	Participants shared personal photos that grounded discussions in real-life experiences, such as family gatherings, special occasions, or recreational activities that individuals participated in. This showed the real-life moments and spaces in which family members found themselves. This reinforced the credibility and depth of the reflections by focusing on the subjective experiences narrated by the participants. “This photo elicitation is very dangerous [meaning thought-provoking] in the sense that it gets people to talk about their life.” (Participant 2)	Offered insight into the significance of routine, memory, traditions, culture, and identity through family photographs.	This approach enabled the data to be more tangible and engaging for both academic and non-academic audiences. This facilitated the process for participants to express their stories verbally and visually.	Participants retained control over what was shown and discussed, maintaining privacy and agency. The method aligned with respectful and ethical engagement. Having the independence to choose their own photo provided them with the opportunity to think about what they would like to share and how in-depth they were willing to discuss their family dynamics.
Object Elicitation	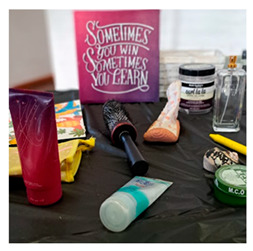 Objects used to relate to family dynamics	The use of meaningful objects (for instance, a hairbrush, Bible, seashell, a medal) opened emotionally rich discussions about family roles and responsibilities, support, love, and struggle. Tangible items anchored the data in lived realities. “It just shows me where I was, and where I’m now, and that’s why I was going to bring [why he brought] the medal.” (Participant 3)	Helped unpack emotions and values in families, what is preserved, honored, or sacrificed, informing both theory and practice in a culturally embedded sense of belonging and care.	Enabled abstract family experiences and relationships to be communicated concretely and memorably to additional participants and researchers.	Participants chose their own objects and stories, allowing for a participant-led pace and depth. This encouraged reflective, personal sharing respectfully. The consent was ongoing and driven by participants.
Painting	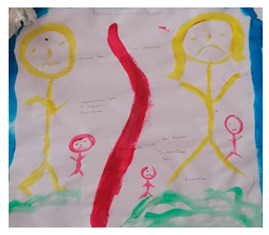 Painting of family household relationships	Enabled the expression of spiritual life, emotional bonds, and family unity. One participant painted hands holding each other to represent support. Another painted family members with a line down the middle, showcasing a broken household. Another painted ‘the change they would like to see’ within their family relations. These artworks offered credible emotional insight.	The paintings provided an opportunity to gain insight into understanding family functioning, with relevance to low-resource contexts where emotion often goes unspoken or ignored. Families were able to relate to one another in terms of hardship (i.e., abuse, neglect), relationships (between parents and parent-child), and communication (for instance, between siblings). “This was actually a therapeutic activity…” (Participant 4)	Emotionally powerful and visually resonant, particularly when showcased with narratives and annotations. For instance, each participant was able to voice their story while the others listened and were able to view their painting.	Provided a non-verbal outlet for reflection; for instance, painting about challenges and trauma was sensitive and self-directed. Participants retained control of meaning and sharing, and thus opted in voluntarily, promoting psychological safety and ethical practice.
LEGO^®^	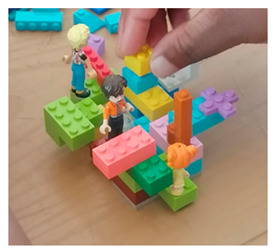 LEGO^®^ interpretation of familial dynamics	Facilitated the construction of household 3D models and shared spaces. Participants’ verbal accounts of their builds revealed power dynamics, roles, and cooperation within families. Through this method, participants were able to externalize relationships and spaces. The act of building supported deeper reflection. “This LEGO^®^ actually made me realize something that I didn’t acknowledge before.” (Participant 5)	Translated abstract family dynamics into visible, modifiable models, which were useful for understanding tensions, aspirations, and coping mechanisms (or the lack thereof) in family life. Common themes such as divorce, abuse, love and respect were heard across both Cape Town and Saldanha Bay Municipality area, ultimately indicating generalizability. “This exercise is to understand how we’re going to get people to think about things differently.” (Participant 6)	This approach was visually communicable among the participants. The models helped explain family structures and routines during dissemination to practitioners and community members. For example, one participant spoke about her house being a home for everyone besides herself and her immediate family. Once this comment was made, additional participants joined the conversation and shared similar sentiments. This ultimately indicates the relatability of the scenarios built with the LEGO^®^ blocks. “These kinds of conversations are very important to stimulate the thinking beyond just our own narrow feelings or psyche.” (Participant 6)	Provided participants with an opportunity to express meaning through creation. This allowed participants to ‘play’ in a safe space using their hands, promoting ethical inclusivity and engagement. “You need to give the child something to play with that also stimulates and challenges their creative thinking.” (Participant 7)
I-Poems	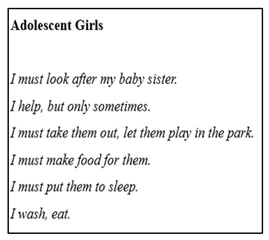 Adolescent I-poem	This approach was constructed directly from interview transcripts, preserving voice, cadence, and emotion. Offered a trustworthy representation of participants’ internal worlds and family relationships. Additionally, the I-poems were shared among the participants to view their read and ‘see’ their lived realities. “This poem, It makes me realize what I have achieved up till now.” (Participant 8)	The I-poems exposed emotional layers (such as hope, fear, respect, responsibility, and care) that often remained unspoken in standard interviews. Participants were able to view their life story in a manner that has never been captured through a poem format before.	The I-poems were lyrical and emotionally resonant, and particularly effective for the participant from which the poem was derived. “Wow, it’s like, when I read it, it’s like…is it really? Is it my life? Is it really me? It overwhelmed me. This poem, it’s like I saw myself in this poem. The life I’m living for my family [crying]. When I spoke about it, it didn’t feel so tough, but when I read it felt like [deep sigh of relief]” (Participant 5)	Participants’ words were used verbatim, maintaining authenticity. Poems were shared with participants for review and consent, respecting voice and ownership. “This helps us understand families, what they do… it’s complex work.” (Participant 9)

## Data Availability

Data is unavailable due to privacy and ethical restrictions.

## Abbreviations

The following abbreviations are used in this manuscript:

HCD	Human Centred Design
LG	Listening Guide
LSP	LEGO^®^ Serious Play
PAR	Participatory Action Research
